# Effects of whole-body vibration on body composition, microbiota, cardiometabolic markers, physical fitness, and quality of life after bariatric surgery: protocol for a randomized controlled trial

**DOI:** 10.1186/s13063-024-08221-7

**Published:** 2024-06-26

**Authors:** Alejandro Gómez-Bruton, Pilar Irún, Angel Matute-Llorente, Gabriel Lozano-Berges, Ana Moradell, Susana Ara-Gimeno, Jorge Subias-Perie, Marta Sánchez-Luengo, Gonzalo Hijos-Mallada, Sandra García-Mateo, Samantha Arechavaleta, María José Palacios Fanlo, Angel Lanas, Jose A. Casajús

**Affiliations:** 1 EXER-GENUD (EXERCISE-Growth, Exercise, NUtrition and Development) Grupo de Investigación, Universidad de Zaragoza, Zaragoza, Spain; 2 Departamento de Fisiatría y Enfermería, Facultad de Ciencias de La Salud y del Deporte, Universidad de Zaragoza, Huesca, Spain; 3 Centro de Investigación Biomédica en Red de Fisiopatología de La Obesidad y Nutrición (CIBEROBN), Instituto de Salud Carlos III, Madrid, Spain; 4 Centro de Investigación Biomédica en Red de Enfermedades Hepáticas y Digestivas (CIBEREHD), Instituto de Salud Carlos III, Madrid, Spain; 5 Instituto de Investigación Sanitaria Aragón (IIS Aragón), Zaragoza, Spain; 6 Facultad de Ciencias de La Salud, Universidad de Zaragoza, Zaragoza, Spain; 7 Service of Digestive Diseases, Hospital Clínico Universitario Lozano Blesa, Zaragoza, Spain; 8 Servicio de Cirugía General y Aparato Digestivo, Hospital Clínico Universitario Lozano Blesa, Zaragoza, Spain; 9 Departamento de Medicina, Psiquiatría y Dermatología, Facultad de Medicina, Universidad de Zaragoza, Zaragoza, Spain; 10 Departamento de Fisiatría y Enfermería, Facultad de Medicina, Universidad de Zaragoza, Zaragoza, Spain

**Keywords:** Morbid obesity, Physical activity, Health, Exercise, RCT

## Abstract

**Background:**

Morbid obesity is a complex chronic condition characterized by a body mass index of 40 kg/m^2^ or higher. The incidence of the condition is on the rise in developed countries, and bariatric surgery has been proposed as a potential solution to address this trend. Nonetheless, bariatric surgery may also result in adverse effects, including a reduction in bone mineral density (BMD) and muscle mass, as well as an increased risk of fractures. The present study aims to elucidate the effects of bariatric surgery and whole-body vibration (WBV) training on body composition, microbiota, physical fitness, quality of life, and cardiometabolic markers.

**Methods:**

Twenty-eight participants (14 females), aged 18 to 50 years, will undergo sleeve gastrectomy surgery. They will be randomly allocated into a control group or a WBV training group. The WBV group will train three times per week with increasing intensities and duration ranging from 30 to 45 min over the 4-month training period. Measurements of body composition (dual-energy X-ray absorptiometry and peripheral quantitative computed tomography), physical fitness (muscular strength, agility, cardiorespiratory fitness, and balance), gait biomechanics, cardiometabolic markers, gut microbiota, quality of life, and physical activity levels will be collected at four different time points: (1) prior to the surgery, (2) 45 days post-surgery, (3) 6 months post-surgery, and (4) 18 months post-surgery.

**Discussion:**

Both groups are expected to experience improvements in most of the aforementioned variables. Nonetheless, we expect the WBV group to show larger improvements proving that the training is effective and safe.

**Trial registration:**

Clinicaltrials.gov NCT05695599. Registered on January 25, 2023.

**Supplementary Information:**

The online version contains supplementary material available at 10.1186/s13063-024-08221-7.

## Introduction

### Background and rationale

Obesity is a multifactorial disease with a prevalence that has tripled since 1980 [1]. By 2025, obesity is projected to increase in 44 European countries, with a described prevalence of obesity of 20% or more in 33 of the 53 European countries [2]. Moreover, the rates of morbid obesity are predicted to increase to 8% and 11% in developed countries such as England and Wales, respectively, by 2035 [3].

The World Health Organization defines morbid obesity as a complex chronic condition characterized by a body mass index (BMI) of 40 kg/m^2^ or higher. It has a significant impact on health, increasing the risk of several comorbidities, affecting the ability to work, and reducing life expectancy by up to 10 years [4]. Consequently, it results in a considerable economic burden on both the individual and society at large. In fact, it has been estimated that European Union countries spend around 7% of their healthcare budgets on treating obesity-related diseases [5, 6].

Although lifestyle changes based on nutrition and physical activity can improve the management of people with obesity, for patients with morbid obesity, surgical treatment through bariatric surgery (BS) has been demonstrated to be more effective and cost-effective than non-surgical measures [7]. BS modifies the composition and functionality of the gut microbiota, thereby reducing the dysbiosis associated with severe obesity and contributing to the remission of obesity-associated comorbidities [8, 9]. The intervention exerts a profound influence on appetite and glucose homeostasis, with gut-derived hormones and the intricate interplay between microbiota, intestine, liver, immune system, and brain playing a pivotal role [10, 11]. The changes in microbiota that occur following BS appear to be approach-specific and overlap with those related to alterations in food habits [12]. While BS is associated with a number of beneficial effects, some types of BS can lead to the malabsorption of calcium and vitamin D, potentially causing adipokine disorders, decrease in bone mineral density (BMD) [13], and an increased risk of fracture in the future [14].

A recent meta-analysis comprising 22 studies evaluating BS patients found that the decrease in BMD was larger in the femoral neck than in the lumbar spine [15]. Additionally, a time-dependent loss of BMD was observed, with larger decreases found in longer follow-up periods. This is consistent with another recent meta-analysis that included 14 studies which evaluated volumetric BMD and bone quality finding negative effects of Roux-en-Y gastric bypass on these variables [16]. The growing evidence of the adverse effects of BS on bone is clear, with the European Calcified Tissue Society publishing a position statement in 2022 stating that BS is associated with a 21–44% higher risk of all fractures [17].

The reduction in BMD may be attributed to a number of factors, including mechanical unloading resulting from a loss of body mass, loss of muscle mass, impaired calcium absorption, changes in hormonal status, and increases in bone marrow adiposity [17]. These factors may also increase the risk of fractures due to alterations in the center of mass, walking biomechanics, and a reduction in muscle mass and strength of the lower limbs. A recent meta-analysis demonstrated that post-surgery exercise interventions can have a positive effect on BMD [18]. In addition, exercise has been shown to have beneficial effects on gut microbiota composition and diversity [19–21] and in reducing inflammation [21, 22]. However, the meta-analysis only included three studies that combined resistance and aerobic training in two to five weekly sessions with an average duration of 45 min per session. Although these interventions are interesting, they require a significant amount of material and space, which makes them difficult to develop. Consequently, shorter and simpler interventions that can be performed in medical clinics and hospitals are needed to maintain participant motivation and avoid the need for a lot of space and equipment.

Whole-body vibration (WBV) training may be a potential solution, as this type of training has demonstrated to be effective in improving body composition and strength in obese patients [23, 24]. Research from our group has shown that it can also improve balance [25] and bone mass [26] in populations with compromised bone mass. In addition, the vibrating platform is only about 1 m^2^, so training can take place in a small space. Therefore, it could be an ideal method for clinics and hospitals to implement.

### Objectives

The objective of the present project is to describe the impact of vertical sleeve gastrectomy (SG) on a range of variables, including body composition (bone, fat, and lean mass), inflammatory, cardiometabolic, liver and bone biomarkers, fecal microbiota, balance and postural control, walking biomechanics, anxiety and depression, levels of physical activity and physical fitness, and quality of life. Furthermore, the study will assess the impact of a 4-month WBV intervention on the aforementioned variables. Finally, the results will enable us to assess the cost-effectiveness of the intervention.

## Material and methods

### Study design, research ethics approval, protocol registration, and reporting

This protocol is a two-arm superiority randomized controlled trial. It has been registered in Clinicaltrials.gov NCT05695599 and approved by the Ethics Committee of Clinical Research of Aragón (CEICA, Spain) with reference number C.I. PI22/380. In the event that changes to the protocol are necessary during its implementation, permission will be sought from the Ethics Committee before applying the changes. Participants will be randomly assigned to one of two groups: (1) the control group (CG) or (2) the WBV group. The study design and this protocol manuscript adhere to the SPIRIT reporting guidelines and the SPIRIT 2013 checklist is presented in Supplementary Table 1 [27]. Moreover, the exercise intervention is explained in accordance with the Consensus on Exercise Reporting Template (CERT): Explanation and Elaboration Statement [28]. The 16 items that should be completed in accordance with the CERT are presented in Supplementary Table 2.

**Fig. 1:**
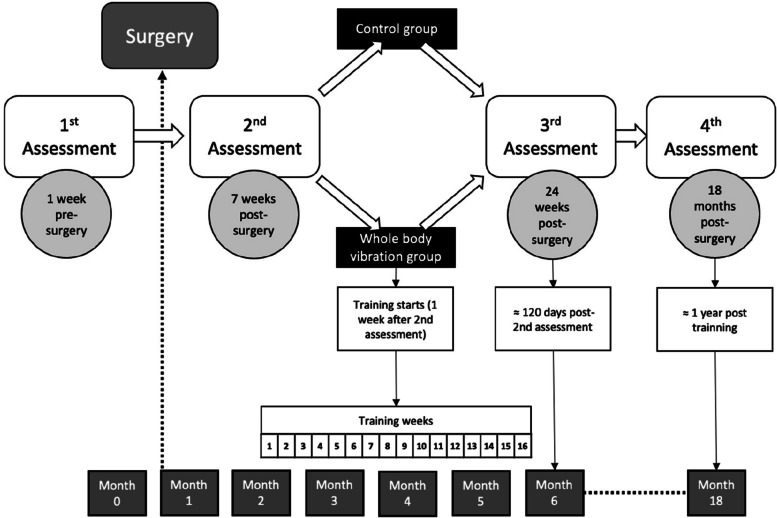
Study timeline

As illustrated in Fig. 1, the study will span a period of 18 months. Four different assessments will be conducted at various intervals: T1 (approximately 1 week before the surgery), T2 (7 ± 1 weeks post-surgery), T3 (24 ± 2 weeks post-surgery), and T4 (18 months ± 2 weeks post-surgery).

Due to the characteristics of the surgery and the expected recruitment difficulties, this study is expected to last a total of 4 years.

### Study setting

The study will be conducted at the public Clinico Lozano Blesa Hospital and the EXER-GENUD (EXERCISE-Growth, Exercise, Nutrition and Development) research group from the University of Zaragoza. Both locations are situated in Zaragoza, Spain.

### Participants

#### Sample size

The sample size for the present study was calculated using G*POWER 3.1. The F-test family, repeated measures, within-between interaction for two groups and four measurements was selected, assuming a correlation among the repeated measurements of 0.5. A total of 12 participants per group were required to achieve a power of 80% with a significance level of 5% under the assumption that we would find small-medium effect sizes (partial eta square of 0.06). This would entail a total of 24 participants. In the event of a 15% attrition rate during the experiment, it would be necessary to recruit an additional 3.6 participants. Consequently, the final study sample would be of 28 participants (14 per group).

#### Eligibility criteria

Inclusion criteria

1. Individuals undergoing a SG with a BMI above 40 kg/m^2^ and a body mass of less than 180 kg
2. Aged between 18 and 50 years

Exclusion criteria

1. Have suffered any type of cancer in the last 5 years
2. Have a pacemaker or have been diagnosed with a cardiac problem that disables the patient from participating in an exercise program
3. Have suffered an acute myocardial infarction in the last 6 months
4. Have recently suffered a fracture
5. Have any other condition that the physician deems as a contraindication for performing supervised physical exercise

#### Recruitment

The recruitment process will be conducted in the Clinico Lozano Blesa Hospital, where each selected participant will engage in an individualized meeting with a nurse. Should they meet the inclusion criteria and decide to participate, they will be required to sign an informed consent. Furthermore, the informed consent form will also require the participants to consent to the collection of biological specimens (stools and blood samples).

### Intervention

#### Randomization, allocation, and blinding

In order to ensure fairness, individuals who meet the eligibility criteria will be randomly assigned (1:1) to either a WBV group or a CG through the webpage https://www.sealedenvelope.com, taking sex into account (1:1). At the second visit, both groups will be provided with a brochure outlining the World Health Organization’s physical activity recommendations, as well as an ad hoc nutritional information sheet. A nurse will be responsible for recruiting all participants and will then direct them to the researchers, who will inform the participants of their group allocation, in accordance with the randomized order. Both groups will receive standard care, but the intervention group will also engage in an exercise intervention as outlined below. Given that the intervention will comprise WBV and that researchers will be involved in it, only the nurse and the statistician performing the statistics will be blinded. There is no foreseeable reason for the statistician to be unblinded to participant allocation.

#### Exercise program rationale

The main aim of the current exercise program is to reduce the loss of bone and lean mass that is commonly observed after BS. Maintaining lean mass is crucial as it helps to preserve the basal metabolic rate, which often declines after BS [29], and may negatively affect body mass loss and body mass maintenance in the long run. Avoiding the loss of lean mass will lead to the preservation of muscle strength, enabling individuals to engage in more intense physical activity and improve their quality of life. Moreover, it is possible that an exercise intervention may also have a positive effect on cardiovascular markers and the gut microbiota.

High-impact exercises that involve jumping and high-speed changes of direction have been demonstrated to be the most effective in improving bone mass [30]. However, these exercises are contraindicated for individuals with obesity, as the additional weight they carry can cause adverse effects on the joints. This is due to the high forces generated during these activities. Therefore, an alternative intervention that has demonstrated efficacy in enhancing bone and lean mass, reducing fat mass, and improving some cardiometabolic markers is training with WBV platforms. A review conducted by Zago et al., which included 18 studies with obese patients, demonstrated that WBV training can improve bone and lean mass [31].

A 16-week training program was selected based on a previous study that included a large number of clinical trials, which found positive effects of exercise interventions that lasted more than 3 months [32]. A 16-week intervention was deemed appropriate, given that bone adaptation requires a longer period than that of lean and fat mass. Although the present protocol includes elastic band training, which could also mask the WBV results, this type of training is only performed for the first 6 weeks. Its purpose is to improve muscle strength in the upper limbs and core, in order to work them out in the next 10 weeks with the WBV platform.

#### Intervention description

##### Supervision and adherence

All training sessions will be overseen by a sports scientist with prior experience in working with WBV platforms. The training will be conducted on a one-to-one basis, with the trainer guiding the participant through each session.

The trainer will contact participants each week via WhatsApp to schedule the upcoming training sessions. The training will be provided free of charge for all participants and will be conducted at the GENUD-Lab (University of Zaragoza, Spain), with no home-based component. The laboratory is equipped with a dedicated space for WBV training and a Smith machine, which will be employed for the attachment of elastic bands and the development of the elastic band training.

During each training session, the trainer will record the following information in each participant’s training book:

- Date and the participant’s attendance at the training session
- Warm-up: Cycling Watts, perceived exertion (using a validated OMNI scale specific for cycle ergometer [33]) and heart rate (using a Polar H10 heart rate sensor)
- WBV training: heart rate and perceived exertion (using a validated visual scale [34] specific for WBV) at the midpoint and conclusion of the training session
- Elastic band training: the color of the elastic band, heart rate, and perceived exertion (using a validated visual scale specific for Thera-Band bands [35])
- Adverse effects during the session (pain, discomfort, etc.)
- Exercises performed by the individual during the previous 72 h outside of the laboratory

At the conclusion of each month, the trainer will evaluate the participant’s attendance percentage and offer constructive feedback to those with a high attendance rate. For those with a low attendance rate, the trainer will discuss potential causes and solutions to improve attendance. In order to progress to the subsequent week, a minimum of two sessions of assistance is required. For instance, should a participant complete only one session in week 5, the following week they will perform one session of week 5 and two sessions of week 6, rather than three sessions of week 6. This adaptation ensures that the increase in intensity is gradual and therefore more manageable. There are no other criteria for discontinuing or modifying allocated interventions. Participants may withdraw voluntary from the trial at any point. They may provide no reason for their withdrawal or may provide any reason they see fit.

All participants will continue with their usual pharmaceutical care during the trial. There is no restriction on accessing external therapy or exercise groups.

##### Session structure

The structure of the sessions will be:

A Warm-up

The warm-up will entail cycling at a frequency between 60 and 70 revolutions per minute. This will be performed on an electromagnetic cycle ergometer (Ergoline Viasprint150p). The initial session will be conducted with a 60-W resistance load. Subsequently, individual adjustments in the watt resistance will be made in order to achieve a moderate activity level (a score of 4–5 on a 0 to 10 scale) on a validated OMNI scale specific to cycle ergometer [33]. Consequently, in the event that a participant indicates a score of less than 4 on the OMNI scale, the resistance will be increased by 5 W in the next session.

B WBV training

**Fig. 2:**
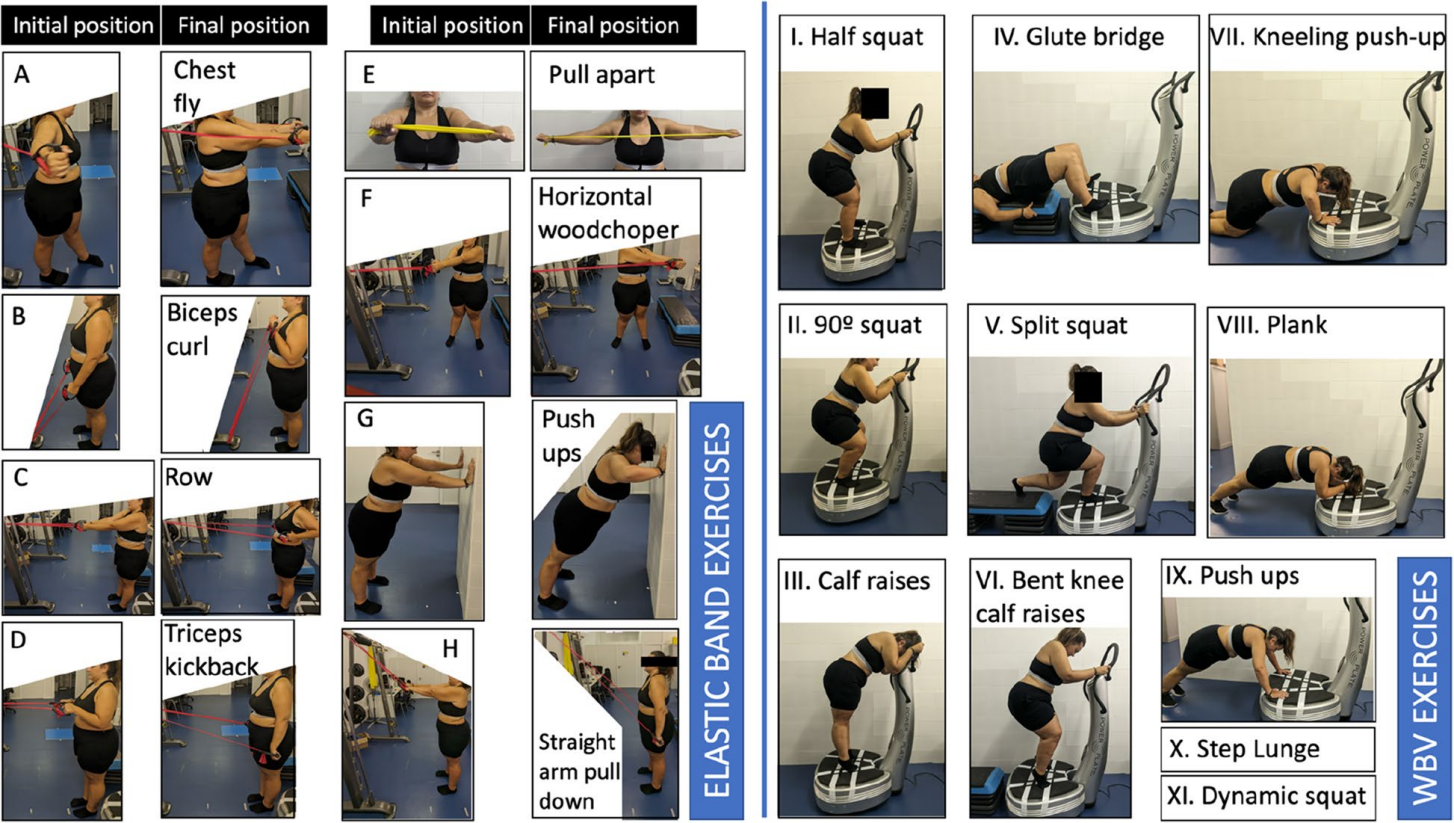
Elastic band and WBV exercises. Exercise X is an exercise that starts with the participant standing on a step and advancing one leg to step on the WBV platform finishing in split squat position (exercise V) to then go back to standing up on the step and repeating with the other leg. Exercise XI is a dynamic squat from 120 to 90°

The WBV will be performed on a synchronous vertical vibration platform (Power Plate® Pro5; PowerPlate, Amsterdam, The Netherlands) with the participant positioned barefoot and with a contoured mat provided by Power Plate®. The WBV protocol is presented in Table 1. The protocol details for months 1–4 can be found in Supplementary Tables 3, 4, 5, and 6, respectively. Prior to commencing the WBV session, the supervisor will provide an explanation of the exercises to be performed during the session. All exercises will be performed with both the forefoot and the heel touching the platform, with the exception of calf raises and bent knee calf raises, which will be performed without contact of the heel with the platform. In order to prevent skidding on the mat provided by the Power Plate, participants will wear socks. As specified in Fig. 2, all exercises will be performed with flexion of at least one lower limb joint in order to prevent vibrations from being transmitted to the head. At the beginning of each month, novel exercises will be introduced, and participants will be allowed to grasp the WBV platform handrail with both hands in order to perform the exercises during the first week. In the subsequent week, participants will be instructed to perform the exercise with one hand only. Over the course of the final weeks of each month, they will be encouraged to progress to a position where they are able to perform the exercises without holding on to the handrail, as detailed in Supplementary Tables 3, 4, 5, and 6. It is important to note that no dumbbells or resistance bands will be utilized during the performance of exercises on the Power Plate®.

**Table 1:** WBV protocol

Week	Warm-up (min)	WBV exercises (repetitions) [duration (s)]	Rest (s)	Freq. (Hz)	Ptp Disp. (mm)	Complimentary exercises^b^	Session duration (min)^d^
1	6	Half squat (× 10) [20 s]	60	30	1	(A, B, C, D) × 1 set × 12 reps	28
2	6	Half squat (× 15) [20 s]	60	30	1	(A, B, C, D) × 2 sets × 12 reps	43
3	8	Half squat (× 12) [20 s]	60^a^	30	1	(A, B, C, D, E, F, G, H) × 3 sets × 12 reps	40
		Calf raises (× 12) [20 s]	60^a^	30	1		
4	8	Half squat (× 10) [30 s]	60^a^	30	1	(A, B, C, D, E, F, G, H) × 3 sets × 12 reps	38
		Calf raises (× 10) [30 s]	60^a^	30	1		
5	10	90° squat (× 10) [30 s]	60^a^	30	1	(A, B, C, D, E, F, G, H) × 3 sets × 12 reps	40
		Calf raises (× 10) [30 s]	60^a^	30	1		
6	10	90° squat (× 10) [30 s]	60^a^	30	1	(A, B, C, D, E, F, G, H) × 3 sets × 12 reps	46
		Calf raises (× 10) [30 s]	60^a^	30	1		
7	10	90° squat (× 10) [30 s]	30	30	1	(G) × 5 sets × 12 reps	42
		Calf raises (× 10) [30 s]	30	30	1		
		Glute bridge (× 5) [15 s]	45	30	1		
8	10	90° squat (× 12) [30 s]	30	30	1	(G) × 2 sets × 12 reps (V) × 2 sets × 15 s (VII) × 3 sets × 15 s	48
		Calf raises (× 12) [30 s]	30	30	1		
		Glute bridge (× 5) [15 s]	45	30	1		
9	10	Split squat (× 10) [30 s]	30	30	1	–	42
		BN calf raises (× 10) [30 s]	30	30	1		
		Kneeling push up (× 4) [15 s]	45	30	1		
		Glute bridge (× 6) [30]	30	30	1		
10	10	Split squat (× 11) [30 s]	30	35	1	(VIII) × 1 set × 10 s	45
		BN calf raises (× 11) [30 s]	30	35	1		
		Kneeling push up (× 4) [30 s]	30	30	1		
		Glute bridge (× 6) [30]	30	30	1		
11	10	Split squat (× 10) [30 s]	30	35	1	–	42
		Plank (× 2) [15 s]	45	30	1		
		BN calf raises (× 10) [30 s]	30	35	1		
		Kneeling push up (× 3) [30 s]	30	30	1		
		Glute bridge (× 5) [30 s]	30	30	1		
12	10	Split squat (× 7) [45 s]	15	35	1	(X) × 2 sets × 10 reps	38
		Plank (× 3) [15 s]	45	30	1		
		BN calf raises (× 7) [45 s]	15	35	1		
		Kneeling push up (× 4) [30 s]	30	30	1		
		Glute bridge (× 5) [30 s]	30	30	1		
13	10	Dynamic squat (× 9) [45 s]	15	30	2	–	39
		Plank (× 4) [15 s]	45	30	1		
		Step lunge (× 5) [30 s]	30	30	1		
		Kneeling push up^c^ (× 4) [15 s]	45	30	1		
		Glute bridge (× 5) [30 s]	30	30	1		
14	10	Dynamic squat (× 10) [45 s]	15	30	2	–	42
		Plank (× 4) [20 s]	40	30	1		
		Step lunge (× 6) [30 s]	30	30	1		
		Kneeling push up^c^ (× 4) [20 s]	40	30	1		
		Glute bridge (× 6) [30 s]	30	30	1		
15	10	Dynamic squat (× 11) [45 s]	15	30	2	–	45
		Plank (× 4) [20 s]	40	30	1		
		Step lunge (× 7) [45 s]	15	30	1		
		Push-ups (× 5) [20 s]	40	30	1		
		Glute bridge (× 6) [30 s]	30	30	1		
16	10	Dynamic squat (× 11) [45 s]	15	30	2	–	45
		Plank (× 4) [20 s]	40	30	1		
		Step lunge (× 7) [45 s]	15	30	1		
		Push-ups (× 5) [20 s]	40	30	1		
		Glute bridge (× 6) [45 s]	15	30	1		

^a^Active rest (elastic band exercises during the rest); ^b^Complimentary exercises explained in Fig. 2; ^c^Feet on a step that is the same height as the WBV so that the push-ups are performed totally horizontal; ^d^Includes a 2-min rest between the warm-up and the beginning of the WBV training; *BN*, bent knee; *Freq.*, frequency; *Ptp Disp.*, peak to peak displacement

As illustrated in Table 1, complementary exercises will be conducted during the initial 6-week period and are elucidated in detail in Fig. 2 and in the “Elastic band training” subsection described below. During certain weeks, the complementary exercises will be performed on the WBV platform without the device being activated as a means of familiarization, while in the following weeks, the same exercises will be performed with the vibration stimulus.

C. Elastic band training

The initial 6-week phase of the WBV training program will be accompanied by an elastic band training component. All exercises will be performed at a speed of 2 s for the concentric phase and 2 s for the eccentric phase. The exercises will be performed using Thera-bands (Thera-Band, Hadamar, Germany). Participants will start with the yellow band and perform the exercises until they reach approximately a 200% elongation at the conclusion of the concentric phase. As participants progress, they will increase the intensity of the exercises using different colors of bands. The tension generated by each band at a 200% elongation is as follows: yellow (1.69 kg-force (kgF)), red (2.26 kgF), green (2.91 kgF), blue (3.52 kgF), black (5.23 kgF), silver (5.91 kgF), and gold (7.55 kgF) [36]. At the conclusion of the final circuit of the day, a visual scale designed for Thera-Band bands [35] will be employed. Should participants indicate a perception below 5 (“somewhat hard”) on a scale of 10, a harder band will be used in the next session.

During the first week, the exercises will be performed after the WBV training. Participants will perform a circuit of four exercises (chest fly, biceps curl, row, and triceps kickback (Fig. 2)) with 12 repetitions per exercise and a 1-min rest interval between each exercise. The circuit will be repeated twice during the second week. The first repetition will be performed after the fifth WBV repetition and the second after the tenth WBV repetition: 5 WBV repetitions, circuit (4 exercises * 12 reps), 5 WBV repetitions, circuit (4 exercises * 12 reps), 5 WBV repetitions.

During the third week, four new exercises will be introduced including pull apart, horizontal woodchopper, wall push-ups, and straight arm pull down (Fig. 2). From week 3 to week 6, the same circuit will be performed three times with 12 repetitions per exercise.

The exercises will be incorporated into the remaining WBV repetitions. This will result in an active rest period of approximately 1 min between the vibration stimulus (the time that participants take to do the elastic band exercises).

##### Training frequency, intensity, and duration

Those allocated to the WBV group will attend three weekly supervised sessions for 16 weeks, with a total of 47 sessions completed (during the first week, only two sessions are performed). Participants will be permitted to select the time of day and day of the week for each session, provided that a minimum of 24 h elapsed between each session and that the training is not performed on three consecutive days.

The duration of the sessions will range from 28 to 46 min, with the intensity increasing weekly through an increase in the number of repetitions, time of vibration, or reduction in rest times as illustrated in Table 1.

In the WBV, all participants will receive the same stimulus, resulting in varying internal loads for each individual. Nevertheless, for the warm-up and the elastic band training, the resistances will be adjusted as previously explained in order to achieve a similar internal load for all participants.

##### Whole-body vibration device

Supplementary Table 7 presents all the information regarding the vibration device that will be used in the present study in accordance with the “*Reporting Guidelines for Whole-Body Vibration Studies in Humans, Animals and Cell Cultures: A Consensus Statement from an International Group of Experts*” guidelines [37]. The specific device presents a constant frequency and magnitude and the vibration parameters have been previously tested in our laboratory [38].

### Outcome variables

The primary outcomes will be (1) body composition, (2) results obtained from the adapted Bruce protocol, (3) basal metabolic rate, and (4) perceived quality of life. All the other variables will be considered as secondary variables. Both primary and secondary outcomes are explained in detail below.

**Fig. 3:**
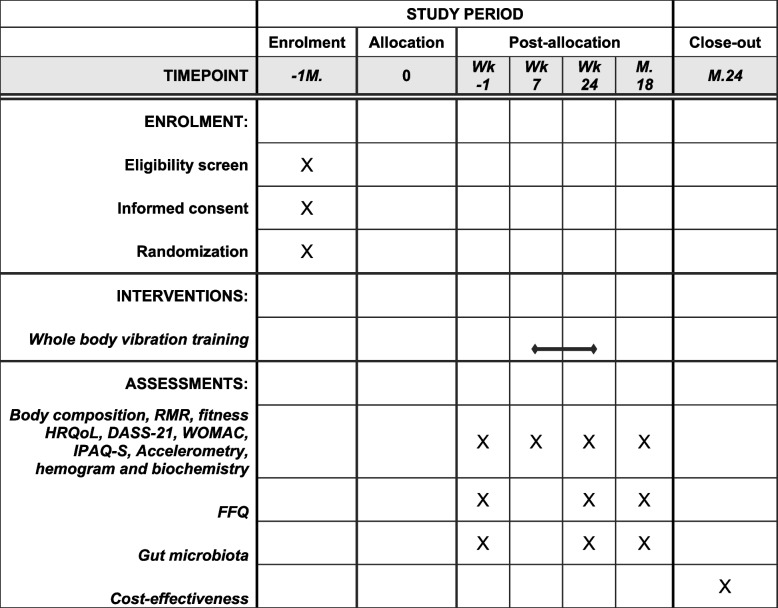
Assessments and intervention timeline. Wk, week; M., month; FFQ, food frequency questionnaire; RMR, resting metabolic rate; WOMAC, the Western Ontario and McMaster questionnaire; IPAQ, International Physical Activity Questionnaire; DASS-21, Depression Anxiety Stress Scale questionnaire; HRQoL, Health-Related Quality of Life

All tests will be conducted in the GENUD-Lab, with the exception of the blood and stool collection, which will be performed at Clinico Lozano Blesa Hospital (situated a 5-min walk from the GENUD-Lab). The outcome variables will be assessed at four different time points (see Fig. 1 or 3). In each time point, participants will attend the laboratory on two different days, with less than a week between them. On each day, different variables will be collected, as detailed below.

#### Day 1

On the first day of the study, participants will attend the laboratory in a fasting condition between 8 and 9 am.

##### General information collection

All data and tests will be collected by sport scientists, with the exception of the food frequency questionnaire, which will be performed by a nutritionist, the exercise testing, which will be performed by a sports and exercise physician, and the collection of blood and stool samples, which will be performed by a nurse. The data collection process will include the following variables: sex, date of birth, mode of transportation to the research center (car, walking, or public transport), level of education, and dominant hand (left or right).

##### Resting metabolic rate (RMR)

RMR will be assessed with a canopy utilizing the Cosmed Quark (Cosmed, Rome, Italy). Participants will lie on a gurney in the supine position for 30 min while wearing a canopy. The data collected during this time will be processed with the Omnia computer software provided by Cosmed. The data collected will include RMR (kcal/day), respiratory quotient, oxygen consumption (VO_2_; ml/min), production of carbon dioxide (VCO_2_; ml/min), and energy obtained from lipids, carbohydrates, and proteins.

##### Anthropometric data

Height and sitting height will be measured without shoes with a stadiometer SECA 225 (SECA, Hamburg, Germany) to the nearest 0.1 cm. Body mass (kg) and body composition (fat and fat-free mass) will be collected with a bioimpedance device (TANITA MC780MA-N, Tanita, Tokyo, Japan).

##### Body composition

A Hologic Horizon QDR dual energy X-ray absorptiometry (DXA) scanner (Hologic Inc, Bedford, MA) will be used to perform whole-body, hip, and lumbar spine scans. The left hip will be measured in all participants. The three scans will yield areal BMD (aBMD, g/cm^2^), bone mineral content (BMC, g), and bone area (cm^2^). The Horizon QDR series enables for a whole-body scan in patients with morbid obesity as the arm and/or leg can be placed outside the scanning area (due to the size limitations of the DXA device, it may not be possible to scan the entire body of large patients). The software will then copy the complete scanned arm or leg to the arm or leg with missing information. In addition to the assessment of aBMD, BMC, and bone area, the whole-body scan will also yield data on lean and fat mass (in grams) and visceral adipose tissue (area in cm^2^, volume in cm^3^, and mass in grams) from the whole-body scan. The trabecular bone score will also be determined using the Hologic-specific software (TBS Hologic).

The bone structure, strength indexes, and BMC of the non-dominant forearm will be measured using a peripheral quantitative computed tomography (pQCT) using a Stratec XCT-2000 L pQCT scanner (Stratec Medizintechnik, Pforzheim, Germany). The forearm will be measured at 4% of the total length to evaluate trabecular bone and at 66% of the total length to evaluate cortical bone. The coefficients of variation for the pQCT in our laboratory have been published elsewhere [39].

Although the International Society of Clinical Densitometry recommends bone scans to be performed with a year difference when a new treatment has begun (in our case the exercise treatment), they also state that in conditions associated with rapid bone loss, testing more frequently is appropriate [40]. It has been demonstrated that BS has a detrimental effect on bone loss, particularly in the initial year following surgery [16]. Previous studies have indicated that significant bone loss can occur as early as 6 months post-surgery [41].

##### The Western Ontario and McMaster (WOMAC) questionnaire, International Physical Activity Questionnaire (IPAQ), and Depression Anxiety Stress Scale questionnaire (DASS-21)

The three questionnaires will be completed by the participants with the assistance of the researchers in an interview format. The WOMAC questionnaire [42] will be used to ascertain the degree of knee and hip pain experienced by the participants. The short version of IPAQ will be used to determine the levels of physical activity and sitting time. The DASS-21 questionnaire will be employed to evaluate depression, anxiety, and stress in the Spanish version [43].

##### Dietary intake

A semiquantitative food frequency questionnaire [44] will be completed at the initial, third, and fourth assessments. The questionnaire comprises 133 items divided into nine food groups: (1) dairy products (15 items); (2) eggs, meat, and fish or seafood (23 items); (3) vegetables (18 items); (4) fruits (16 items); (5) legumes and cereals (11 items); (6) oils and fats (11 items); (7) pastries, cakes, or sweets (13 items); (8) a miscellaneous group (12 items); and (9) drinks (14 items). The questionnaire will refer to the preceding year and will be completed through an interview with a nutritionist. Each item included is accompanied by a description of the typical portion size. The participants will select the frequency of consumption between nine options (ranging from never to six or more times per day). Nutrient intake scores will be calculated using an ad hoc computer program designed for this purpose [45]. This will involve multiplying the frequency of consumption by the nutrient composition of a specified portion size. The selected frequency item will be converted to a daily intake. For example, if a response is 5–6 times a week, it will be converted to 0.78 servings per day. Portion size will be multiplied by the frequency of consumption in order to obtain the daily intake. The data extracted from this questionnaire will comprise total mean energy intake (kcal/day), macronutrients (protein, fat, and carbohydrates in g/day and % kcal of the total macronutrient energy distribution), alcohol (g and % kcal of the total macronutrient energy distribution), types of fatty acids (g/day), types of polyunsaturated fatty acids (PUFA) (n-3 and n-6) (g/day), and vitamins and minerals (mg or µg/day as corresponding to the nutrient in question).

##### Fecal microbiota analysis

The microbial composition will be determined through the use of Shallow Shotgun Metagenome sequencing [46, 47]. In this context, participants will bring to the hospital a stool sample collected at home in the first, third, and fourth assessments. The samples will be placed in a DNA/RNA Shield Fecal collection tube from Zymo Research, which is appropriate for the collection of a microbial snapshot of the sample, thus ensuring the safety, readinees for transport, and stability of the samples at ambient temperature. Samples will be frozen after reception for longer-term storage. Subsequently, DNA will be extracted from the stool samples using the ZymoBIOMICS DNA Miniprep Kit (Zymo Research, USA). Libraries will be constructed and analyzed for DNA samples that meet the requisite quality standards. The qualified libraries will be sequenced with the Illumina PE150 (5 G raw data per sample). The ulterior analysis will include species annotation with Kraken [48], the relative abundance of different species at different taxonomic levels, dimensionality reduction analysis based on species abundance and on the Bray–Curtis dissimilarity of species abundance, sample clustering analysis and anosim analysis, all based on species abundance, metastat [49] and LEfSe analysis [50] on inter-group differential species.

##### Hospital visits

Anthropometric parameters including body mass (SECA 813), height (Sayol stadiometer), waist circumference, hip circumference, and body mass index will be measured in the hospital. Vital signs including arterial blood pressure (Nellcor N5600) will also be recorded. Any chronic diseases and any acute disease that occurred during the 6 months preceding the onset of the study and any pharmacological treatment or nutritional supplement taken during the previous month will be recorded. Furthermore, all participants will undergo a blood extraction to evaluate the following parameters: hemogram and biochemistry, coagulation indicators, iron metabolism, liver enzymes, bone metabolic markers, C-reactive protein, and interleukin 6, in accordance with standard hospital procedures. In addition, tumor necrosis factor-alpha (TNF-α) levels will be quantified by enzyme-linked assay (ELISA) methodology (Quantikine High Sensitivity Human by R&D Systems, Minneapolis, MN, USA).

#### Day 2

##### Static balance—body sway

Participants will be required to stand barefoot on a three-axis force plate (Kistler Type 9260AA; Kistler Instruments Ltd, Hampshire, UK) in three different positions: with their feet parallel to each other, with one foot slightly in front of the other (semi-tandem), and with one foot directly in front of the other (tandem position) [51]. All tests will be conducted twice with the eyes open and twice with the eyes closed, each lasting for 30 s. The data will be processed and exported using the Kistler Mars software. The sway path (measured in millimeters) and velocity (measured in millimeters per second) will be used for further analysis.

##### Walking speed and step analysis

The participants will be requested to walk at their usual pace, with no footwear. To ascertain the time taken by the participants to complete the 6-m distance, photocells (Witty Gate Wireless Training Timer Photocells, Microgate, Italy) with a precision of 0.001 s will be placed at a distance of 6 m apart. Furthermore, the time taken to complete the 4-m distance will also be recorded manually. A two-step gait initiation protocol [52] will be employed with all participants commencing with their left leg. This ensures that the second step, which is performed with the right leg, is on a force plate (Kistler Type 9260AA; Kistler Instruments Ltd, Hampshire, UK) embedded in the walkway. The participants will complete five trials. During the follow-up visits, the tests will be performed at the participant’s normal speed (five attempts) and at the same speed as they performed the initial evaluation (mean of three attempts (excluding the fastest and slowest attempts)). A trial will be accepted if the speed is within a margin of ± 5% of the target speed. The Kistler Mars software will be used to process and export the data. The software provides more than 20 variables, which will be used to analyze the data. These include time parameters (stance, braking, and propulsion times), vertical component parameters, anterior–posterior component parameters, medio-lateral component parameters, and center of pressure parameters.

##### Timed Up and Go (TUG) test

The TUG [53] test will be performed twice with shoes. Participants commence the test seated on a chair and are instructed to stand up, walk a distance of 3 m, turn around, walk back to the chair, and sit down at their maximal speed but without running. The time taken to complete the test (s) will be recorded.

##### Five times sit-to-stand test

Participants will be asked to complete five repetitions of the sit-to-stand movement as rapidly as possible [54]. The time to complete the test (s) and power performed by each participant will be registered.

##### Health-Related Quality of Life (HRQoL) and pain questionnaires

The HRQoL questionnaire will be assessed with the Spanish version of the EUROQOL-5D-5L [55] and the SF-36 [56] questionnaires. The EUROQOL-5D-5L comprises five principal dimensions, 3 associated with physical capacity (mobility, self-care, and usual activities) and two with mental health (pain/discomfort and anxiety/depression). The visual analogic scale ranging from 0 to 100 will also be used. The SF-36 questionnaire encompasses eight health domains and is one of the most used questionnaires to assess HRQoL in patients who undergo BS [57].

##### Modified Bruce protocol

The modified Bruce protocol [58] will be performed on an instrumented treadmill (h/p/cosmos, Nussdorf–Traunstein, Germany). Participants will breathe through a low–dead space mask, with air sampled at 60 ml·min^−1^. Before each test, two-point calibrations of the gas sensors will be completed, using a known gas mixture (16% O_2_ and 5% CO_2_) and ambient air. The ventilatory volume will be calibrated using a 3-l (± 0.4%) syringe. The subjects will wear an instrumented portable electrocardiogram during all the tests (Cosmed Quark T12x). Gas exchange measurements will be registered breath-by-breath during the test and for 3 min of the recovery (Cosmed Quark, Rome, Italy). The objective of this test is to determine the V̇O_2_ peak (defined as the highest values obtained during the test) and the individual ventilatory anaerobic threshold (IAT), which is determined through visual assessment by an experienced sports and exercise physician.

##### Accelerometer

Participants will receive an accelerometer ActiGraph GT9X Link (ActiGraph LLC, Pensacola, FL, USA) and wear it for 7 consecutive days on the non-dominant wrist. Sleep time, sedentary time, and physical activity will be registered with the accelerometer.

### Cost-effectiveness analysis

The economic analysis will be conducted from the perspective of the healthcare system with the objective of estimating the approximate cost of implementing this exercise program for this population. This analysis will include healthcare utilization and intervention costs. The health system cost will be recorded, including the visits of physicians and nurses in primary care, specialist physicians, physiotherapists, social workers, occupational therapists, and dietitian services; medication use; emergency rooms; hospital days; diagnostic imaging tests; blood tests; and metagenomics. Furthermore, the costs of the intervention will be estimated based on records of the time spent by exercise instructors, the use of sports facilities, and the purchase of exercise equipment. The time frame for the analysis will be approximately 6 months.

Two types of economic analysis will be conducted: an intention-to-treat and a per-protocol analysis. The intention-to-treat analysis will include all participants who will complete the trial, whereas the per-protocol analysis will include only those participants who will attend at least 70% of the exercise sessions.

To calculate the incremental cost-effectiveness ratio (ICER), we will examine the differences in costs and effectiveness between the intervention and control groups. Specifically, the incremental cost per participant will be divided by the incremental effectiveness to get the ICER. The ICER will be calculated for the incremental cost per person required to improve the health-related quality of life (HRQOL, assessed through Euroqol-5D [EQ-5D]) and functional capacity (assessed through Short Physical Performance Battery [SPPB]), and reduction of excess weight loss (%) and fat mass (%).

Finally, non-parametric bootstrap techniques of costs and health outcomes will be employed to estimate the probability that an exercise intervention would be cost-effective given a decision-maker’s willingness to pay for each additional unit of health outcome gained, or excess weight loss and fat mass reduced. Furthermore, cost-effectiveness acceptability curves will be presented, illustrating the probability of exercise intervention in this population could be cost-effective compared with the control group.

### Data collection and management

Trained researchers will collect data from both the Clinico Lozano Blesa Hospital and the GENUD-Lab. All information collected during this trial will always be protected and securely stored by the lead researcher. The lead researcher will assign a secure locker in his office to store all investigation documents. Additionally, all electronic material will be safely stored and backed up on the researcher’s computer equipment with a secure password.

### Statistical methods

The Shapiro–Wilk test will be used to test for normality of the data while variance homogeneity and sphericity will be assessed with the Levene and Mauchly tests, respectively. To assess changes over time in the assessed outcomes, a repeated-measures ANOVA with Bonferroni correction will be used to assess the interaction between group (control group vs. WBV) and time (baseline vs. 2nd vs. 3rd vs. 4th assessments). In the absence of a normal distribution, data will be analyzed with the Wilcoxon test for within-group comparisons and the Mann–Whitney test for between-group comparisons using Bonferroni’s correction. To test for associations between variables, both correlations and linear regressions will be performed. The significance level will be set at 5%. Per-protocol and intention-to-treat analyses will be performed in order to account for the possible dropouts during the study. In the per-protocol analysis, we will include only the participants who attend at least 70% of the sessions and undergo the baseline and post-intervention outcome assessments. For the intention-to-treat analysis, we will consider all the participants who will undergo the baseline assessments and subsequently participate in at least one additional assessment following their group assignment will be considered.

## Discussion

The prevalence of morbid obesity is rising in developed countries [3] and BS is one of the most effective interventions for combating this condition. Nonetheless, this treatment is associated with some adverse effects that could be mitigated by exercise.

Our study aims to investigate the impact of BS on body composition, physical fitness, microbiota, and quality of life among other factors. Furthermore, we will assess whether a WBV intervention can help to mitigate the decline in bone and lean mass caused by BS, while also improving quality of life. Previous studies with obese patients [31] indicate that the intervention may be effective and well-tolerated by participants. Finally, we will evaluate the cost-effectiveness of the exercise intervention in order to determine its future viability. Should the cost-effectiveness results be positive, researchers would seek for funding to extend the study in time (5- and 10-year follow-up) and number of participants. Moreover, they would attempt to include the exercise intervention as part of the usual care of the Spanish national health system.

## Conclusions

The present study will investigate the impact of WBV training on patients who have undergone SG. It is hypothesized that a 16-week WBV training program will help to prevent the loss of lean and bone mass that can result from BS. Moreover, the basal metabolic rate will also be maintained, which will result in a higher loss of fat mass and lower body mass recovery in the future. A cost-effectiveness analysis will also be conducted to assess the economic utility of this approach. This study will be the first of its kind to explore the effects of WBV training in this particular group of patients.

### Supplementary Information

Supplementary Material 1.
