# Supplementary material for: Effects of whole-body vibration on body composition, microbiota, cardiometabolic markers, physical fitness, and quality of life after bariatric surgery: protocol for a randomized controlled trial
Source: Trials. 2024 Jun 26;25:413. doi: 10.1186/s13063-024-08221-7 (PMC11210142; doi:10.1186/s13063-024-08221-7)
Supplement: Supplementary file 1 — Supplementary Material 1. [file 13063_2024_8221_MOESM1_ESM.docx]

# **Supplementary Table 1.** SPIRIT Checklist for Trials

|  |  | **Reporting Item** | **Page and Line Number** |
| --- | --- | --- | --- |
| **Administrative information** | | | |
| Title | [#1](https://www.goodreports.org/reporting-checklists/spirit/info/#1) | Descriptive title identifying the study design, population, interventions, and, if applicable, trial acronym | Page 1, line 1 |
| Trial registration | [#2a](https://www.goodreports.org/reporting-checklists/spirit/info/#2a) | Trial identifier and registry name. If not yet registered, name of intended registry | Page 2, Line 37 |
| Trial registration: data set | [#2b](https://www.goodreports.org/reporting-checklists/spirit/info/#2b) | All items from the World Health Organization Trial Registration Data Set | Page 4, Line36 |
| Protocol version | [#3](https://www.goodreports.org/reporting-checklists/spirit/info/#3) | Date and version identifier | Page 18, line 11 |
| Funding | [#4](https://www.goodreports.org/reporting-checklists/spirit/info/#4) | Sources and types of financial, material, and other support | Page 18, line18 |
| Roles and responsibilities: contributorship | [#5a](https://www.goodreports.org/reporting-checklists/spirit/info/#5a) | Names, affiliations, and roles of protocol contributors | Page 19, line 1 |
| Roles and responsibilities: sponsor contact information | [#5b](https://www.goodreports.org/reporting-checklists/spirit/info/#5b) | Name and contact information for the trial sponsor | Page 18, line 26 |
| Roles and responsibilities: sponsor and funder | [#5c](https://www.goodreports.org/reporting-checklists/spirit/info/#5c) | Role of study sponsor and funders, if any, in study design; collection, management, analysis, and interpretation of data; writing of the report; and the decision to submit the report for publication, including whether they will have ultimate authority over any of these activities | Page 18, line 20 |
| Roles and responsibilities: committees | [#5d](https://www.goodreports.org/reporting-checklists/spirit/info/#5d) | Composition, roles, and responsibilities of the coordinating centre, steering committee, endpoint adjudication committee, data management team, and other individuals or groups overseeing the trial, if applicable (see Item 21a for data monitoring committee) | Page 18, line 31 |
| **Introduction** |  |  |  |
| Background and rationale | [#6a](https://www.goodreports.org/reporting-checklists/spirit/info/#6a) | Description of research question and justification for undertaking the trial, including summary of relevant studies (published and unpublished) examining benefits and harms for each intervention | Page 3, lines 3-60 |
| Background and rationale: choice of comparators | [#6b](https://www.goodreports.org/reporting-checklists/spirit/info/#6b) | Explanation for choice of comparators | Page 6, line 31 |
| Objectives | [#7](https://www.goodreports.org/reporting-checklists/spirit/info/#7) | Specific objectives or hypotheses | Page 4, line 22 |
| Trial design | [#8](https://www.goodreports.org/reporting-checklists/spirit/info/#8) | Description of trial design including type of trial (eg, parallel group, crossover, factorial, single group), allocation ratio, and framework (eg, superiority, equivalence, non-inferiority, exploratory) | Page 4, line 36 |
| Study setting | [#9](https://www.goodreports.org/reporting-checklists/spirit/info/#9) | Description of study settings (eg, community clinic, academic hospital) and list of countries where data will be collected. Reference to where list of study sites can be obtained | Page 5, line 33 |
| Eligibility criteria | [#10](https://www.goodreports.org/reporting-checklists/spirit/info/#10) | Inclusion and exclusion criteria for participants. If applicable, eligibility criteria for study centres and individuals who will perform the interventions (eg, surgeons, psychotherapists) | Page 5, line 54 |
| Interventions: description | [#11a](https://www.goodreports.org/reporting-checklists/spirit/info/#11a) | Interventions for each group with sufficient detail to allow replication, including how and when they will be administered | Page 6, line 18 |
| Interventions: modifications | [#11b](https://www.goodreports.org/reporting-checklists/spirit/info/#11b) | Criteria for discontinuing or modifying allocated interventions for a given trial participant (eg, drug dose change in response to harms, participant request, or improving / worsening disease) | Page 7, line 60 |
| Interventions: adherance | [#11c](https://www.goodreports.org/reporting-checklists/spirit/info/#11c) | Strategies to improve adherence to intervention protocols, and any procedures for monitoring adherence (eg, drug tablet return; laboratory tests) | Page 7, line 51 |
| Interventions: concomitant care | [#11d](https://www.goodreports.org/reporting-checklists/spirit/info/#11d) | Relevant concomitant care and interventions that are permitted or prohibited during the trial | Page 8, line 3 |
| Outcomes | [#12](https://www.goodreports.org/reporting-checklists/spirit/info/#12) | Primary, secondary, and other outcomes, including the specific measurement variable (eg, systolic blood pressure), analysis metric (eg, change from baseline, final value, time to event), method of aggregation (eg, median, proportion), and time point for each outcome. Explanation of the clinical relevance of chosen efficacy and harm outcomes is strongly recommended | Page 12, line 1 |
| Participant timeline | [#13](https://www.goodreports.org/reporting-checklists/spirit/info/#13) | Time schedule of enrolment, interventions (including any run-ins and washouts), assessments, and visits for participants. A schematic diagram is highly recommended (see Figure) | Page 5, Figure 1 |
| Sample size | [#14](https://www.goodreports.org/reporting-checklists/spirit/info/#14) | Estimated number of participants needed to achieve study objectives and how it was determined, including clinical and statistical assumptions supporting any sample size calculations | Page 5, line 44 |
| Recruitment | [#15](https://www.goodreports.org/reporting-checklists/spirit/info/#15) | Strategies for achieving adequate participant enrolment to reach target sample size | Page 6, line 15 |
| Allocation: sequence generation | [#16a](https://www.goodreports.org/reporting-checklists/spirit/info/#16a) | Method of generating the allocation sequence (eg, computer-generated random numbers), and list of any factors for stratification. To reduce predictability of a random sequence, details of any planned restriction (eg, blocking) should be provided in a separate document that is unavailable to those who enrol participants or assign interventions | Page 6, line 32 |
| Allocation concealment mechanism | [#16b](https://www.goodreports.org/reporting-checklists/spirit/info/#16b) | Mechanism of implementing the allocation sequence (eg, central telephone; sequentially numbered, opaque, sealed envelopes), describing any steps to conceal the sequence until interventions are assigned | Page 6, line 36 |
| Allocation: implementation | [#16c](https://www.goodreports.org/reporting-checklists/spirit/info/#16c) | Who will generate the allocation sequence, who will enrol participants, and who will assign participants to interventions | Page 6, line 32 |
| Blinding (masking) | [#17a](https://www.goodreports.org/reporting-checklists/spirit/info/#17a) | Who will be blinded after assignment to interventions (eg, trial participants, care providers, outcome assessors, data analysts), and how | Page 6, line 38 |
| Blinding (masking): emergency unblinding | [#17b](https://www.goodreports.org/reporting-checklists/spirit/info/#17b) | If blinded, circumstances under which unblinding is permissible, and procedure for revealing a participant’s allocated intervention during the trial | Page 6, line 38 |
| Data collection plan | [#18a](https://www.goodreports.org/reporting-checklists/spirit/info/#18a) | Plans for assessment and collection of outcome, baseline, and other trial data, including any related processes to promote data quality (eg, duplicate measurements, training of assessors) and a description of study instruments (eg, questionnaires, laboratory tests) along with their reliability and validity, if known. Reference to where data collection forms can be found, if not in the protocol | Page 12,  Study instruments explained in outcome variables section |
| Data collection plan: retention | [#18b](https://www.goodreports.org/reporting-checklists/spirit/info/#18b) | Plans to promote participant retention and complete follow-up, including list of any outcome data to be collected for participants who discontinue or deviate from intervention protocols | Page 18, line 27 |
| Data management | [#19](https://www.goodreports.org/reporting-checklists/spirit/info/#19) | Plans for data entry, coding, security, and storage, including any related processes to promote data quality (eg, double data entry; range checks for data values). Reference to where details of data management procedures can be found, if not in the protocol | Page 17, line 16 |
| Statistics: outcomes | [#20a](https://www.goodreports.org/reporting-checklists/spirit/info/#20a) | Statistical methods for analysing primary and secondary outcomes. Reference to where other details of the statistical analysis plan can be found, if not in the protocol | Page 17, line 26 |
| Statistics: additional analyses | [#20b](https://www.goodreports.org/reporting-checklists/spirit/info/#20b) | Methods for any additional analyses (eg, subgroup and adjusted analyses) | Page 17, line 39 |
| Statistics: analysis population and missing data | [#20c](https://www.goodreports.org/reporting-checklists/spirit/info/#20c) | Definition of analysis population relating to protocol non-adherence (eg, as randomised analysis), and any statistical methods to handle missing data (eg, multiple imputation) | Page 17, line 39 |
| Data monitoring: formal committee | [#21a](https://www.goodreports.org/reporting-checklists/spirit/info/#21a) | Composition of data monitoring committee (DMC); summary of its role and reporting structure; statement of whether it is independent from the sponsor and competing interests; and reference to where further details about its charter can be found, if not in the protocol. Alternatively, an explanation of why a DMC is not needed | Page 18, line 37 |
| Data monitoring: interim analysis | [#21b](https://www.goodreports.org/reporting-checklists/spirit/info/#21b) | Description of any interim analyses and stopping guidelines, including who will have access to these interim results and make the final decision to terminate the trial | Page 18, line 37 |
| Harms | [#22](https://www.goodreports.org/reporting-checklists/spirit/info/#22) | Plans for collecting, assessing, reporting, and managing solicited and spontaneously reported adverse events and other unintended effects of trial interventions or trial conduct | Page 7, line 50  Page 18, line 44 |
| Auditing | [#23](https://www.goodreports.org/reporting-checklists/spirit/info/#23) | Frequency and procedures for auditing trial conduct, if any, and whether the process will be independent from investigators and the sponsor | Page 19, line 29 |
| Research ethics approval | [#24](https://www.goodreports.org/reporting-checklists/spirit/info/#24) | Plans for seeking research ethics committee / institutional review board (REC / IRB) approval | Page 4, line 38 |
| Protocol amendments | [#25](https://www.goodreports.org/reporting-checklists/spirit/info/#25) | Plans for communicating important protocol modifications (eg, changes to eligibility criteria, outcomes, analyses) to relevant parties (eg, investigators, REC / IRBs, trial participants, trial registries, journals, regulators) | Page 4, line 39 |
| Consent or assent | [#26a](https://www.goodreports.org/reporting-checklists/spirit/info/#26a) | Who will obtain informed consent or assent from potential trial participants or authorised surrogates, and how (see Item 32) | Page 6, line 19 |
| Consent or assent: ancillary studies | [#26b](https://www.goodreports.org/reporting-checklists/spirit/info/#26b) | Additional consent provisions for collection and use of participant data and biological specimens in ancillary studies, if applicable | Page 6, line 19 |
| Confidentiality | [#27](https://www.goodreports.org/reporting-checklists/spirit/info/#27) | How personal information about potential and enrolled participants will be collected, shared, and maintained in order to protect confidentiality before, during, and after the trial | Page 18, line 18 |
| Declaration of interests | [#28](https://www.goodreports.org/reporting-checklists/spirit/info/#28) | Financial and other competing interests for principal investigators for the overall trial and each study site | Page 18, line 50 |
| Data access | [#29](https://www.goodreports.org/reporting-checklists/spirit/info/#29) | Statement of who will have access to the final trial dataset, and disclosure of contractual agreements that limit such access for investigators | Page 18, line 23 |
| Ancillary and post trial care | [#30](https://www.goodreports.org/reporting-checklists/spirit/info/#30) | Provisions, if any, for ancillary and post-trial care, and for compensation to those who suffer harm from trial participation | Page 18, line 43 |
| Dissemination policy: trial results | [#31a](https://www.goodreports.org/reporting-checklists/spirit/info/#31a) | Plans for investigators and sponsor to communicate trial results to participants, healthcare professionals, the public, and other relevant groups (eg, via publication, reporting in results databases, or other data sharing arrangements), including any publication restrictions | Page 18, line 27 |
| Dissemination policy: authorship | [#31b](https://www.goodreports.org/reporting-checklists/spirit/info/#31b) | Authorship eligibility guidelines and any intended use of professional writers | Page 19, line 49 |
| Dissemination policy: reproducible research | [#31c](https://www.goodreports.org/reporting-checklists/spirit/info/#31c) | Plans, if any, for granting public access to the full protocol, participant-level dataset, and statistical code | Page 19, line 36 |
| Informed consent materials | [#32](https://www.goodreports.org/reporting-checklists/spirit/info/#32) | Model consent form and other related documentation given to participants and authorised surrogates | Page 19, line 42 |
| Biological specimens | [#33](https://www.goodreports.org/reporting-checklists/spirit/info/#33) | Plans for collection, laboratory evaluation, and storage of biological specimens for genetic or molecular analysis in the current trial and for future use in ancillary studies, if applicable | Page 14, line 46 |

| **Supplementary Table 2.** Consensus on Exercise Reporting Template word | |
| --- | --- |
| 1  Detailed description of the type of exercise equipment | **3.4.3.2. Session structure – Page 8 Line 12** |
| 2  Detailed description of the qualifications, expertise and/or training | **3.4.3.1. Supervision and adherence- Page 7 Line 23** |
| 3  Describe whether exercises are performed individually or in a group | **3.4.3.1. Supervision and adherence- Page 7 Line 27** |
| 4  Describe whether exercises are supervised or unsupervised; how they are delivered | **3.4.3.1. Supervision and adherence- Page 7 Line 27** |
| 5  Detailed description of how adherence to exercise is measured and reported | **3.4.3.1. Supervision and adherence- Page 7 Line 56** |
| 6  Detailed description of motivation strategies | **3.4.3. Intervention description – Page 7 Line 55** |
| 7a  Detailed description of the decision rule(s) to determining exercise progression | **3.4.3.2. Session structure – Page 8 Line 13** |
| 7b  Detailed description of how the exercise program was progressed | **Table 1**  **Supplementary Tables 1, 2, 3 and 4** |
| 8  Detailed description of each exercise to enable replication | **Supplementary Tables 1, 2, 3 and 4**  **Figure 2** |
| 9  Detailed description of any home programme component | **3.4.3.1. Supervision and adherence – Page 7 Line 32** |
| 10  Describe whether there are any non-exercise components | **3.4.1. Randomization – Page 6 Line 27** |
| 11  Describe the type and number of adverse events that occur  during exercise | **3.4.3.1. Supervision and adherence – Page 7 Line 50** |
| 12  Describe the setting in which the exercises are performed | **3.4.3. Intervention description – Page 7 Line 32** |
| 13  Detailed description of the exercise intervention | **3.4.3. Intervention description**  **Table 1**  **Figure 2** |
| 14a  Describe whether the exercises are generic (one size fits all) or  tailored | **3.4.3.2. Session structure**  **3.4.3.3. Training frequency, intensity and duration** |
| 14b  Detailed description of how exercises are tailored to the individual | **3.4.3.2. Session structure** |
| 15 Describe the decision rule for determining the starting level | **3.4.3.2. Session structure – Page 8, line 15** |
| 16a  Describe how adherence or fidelity is assessed/measured | **3.4.3.1. Supervision and adherence - Page 7, line 37** |
| 16b  Describe the extent to which the intervention was delivered as planned | **Not applicable** |

**Supplementary Table 3.** Month 1. Warm up of 6 minutes on week 1 and 2 and of 8 minutes of weeks 3 and 4. Rep.: Repetitions; Freq: Frequency; Disp.: Displacement; Dur.: Duration; Vib.: Vibration; p: Peak; accel: acceleration, RMS: Root mean square.

| **Week** | **Position** | **Hands and arms** | **Feet** | **Rep.** | **Freq. (Hz)** | **Displ. (mm)** | **Dur. (s)** | **Rest (s)** | **Active rest** | **Total vib. (s)** | **Total vib. (min)** | **P. accel (apeak) g** | **P. accel (apeak) m/s^2^** | **RMS Accel. (aRMS) m/s^2^** | **Density (Work/rest)** | **Dose (vibration (g)*time (s))** | **Total weekly dose** | **Acumulated dose** |
| --- | --- | --- | --- | --- | --- | --- | --- | --- | --- | --- | --- | --- | --- | --- | --- | --- | --- | --- |
| **Week 1** | **Half squat** | **Both hands hold** | **normal stance** | **10** | **30** | **1** | **20** | **60** | **NO** | **200** | **3.3** | **1.81** | **17.8** | **12.6** | **0.33** | **362.2** | **1086.6** | **1086.6** |
| **Week 2** | **Half squat** | **One hand hold** | **normal stance** | **15** | **30** | **1** | **20** | **60** | **NO** | **300** | **5** | **1.81** | **17.8** | **12.6** | **0.33** | **543.3** | **1629.9** | **2716.4** |
| Week 3 | Half squat | Crossed arms (Without hold) | wide stance | 12 | 30 | 1 | 20 | 60 | YES | 240 | 4 | 1.81 | 17.8 | 12.6 | 0.33 | 434.6 |  |  |
|  | Calf raises | Both hands hold | normal stance | 12 | 30 | 1 | 20 | 60 | YES | 240 | 4 | 1.81 | 17.8 | 12.6 | 0.33 | 434.6 |  |  |
| **Total week 3** |  |  |  | **24** |  |  |  |  |  | **480** | **8** |  |  |  |  | **869.3** | **2607.8** | **5324.2** |
| Week 4 | Half squat | Crossed arms (Without hold) | wide stance | 10 | 30 | 1 | 30 | 60 | YES | 300 | 5 | 1.81 | 17.8 | 12.6 | 0.5 | 543.3 |  |  |
|  | Calf raises | Both hands hold | normal stance | 10 | 30 | 1 | 30 | 60 | YES | 300 | 5 | 1.81 | 17.8 | 12.6 | 0.5 | 543.3 |  |  |
| **Total week 4** |  |  |  | **20** |  |  |  |  |  | **600** | **10** |  |  |  |  | **1086.6** | **3259.7** | **8583.9** |

| **Week** | **Position** | **Hands and arms** | **Feet** | **Rep.** | **Freq. (Hz)** | **Displ. (mm)** | **Dur. (s)** | **Rest (s)** | **Active rest** | **Total vib. (s)** | **Total vib. (min)** | **P. accel (apeak) g** | **P. accel (apeak) m/s^2^** | **RMS Accel. (aRMS) m/s^2^** | **Density (Work/rest)** | **Dose (vibration (g)*time (s))** | **Total weekly dose** | **Acumulated dose** |
| --- | --- | --- | --- | --- | --- | --- | --- | --- | --- | --- | --- | --- | --- | --- | --- | --- | --- | --- |
| Week 5 | 90º Degree Squat | Both hands hold | normal stance | 10 | 30 | 1 | 30 | 60 | YES | 300 | 5 | 1.81 | 17.8 | 12.6 | 0.5 | 543.3 |  |  |
|  | Calf raises | Both hands hold | normal stance | 10 | 30 | 1 | 30 | 60 | YES | 300 | 5 | 1.81 | 17.8 | 12.6 | 0.5 | 543.3 |  |  |
| **Total week 5** |  |  |  | **20** |  |  |  |  |  | **600** | **10** |  |  |  |  | **1086.6** | **3259.7** | **11843.6** |
| Week 6 | 90º Degree Squat | One hand hold | normal stance | 12 | 30 | 1 | 30 | 60 | YES | 360 | 6 | 1.81 | 17.8 | 12.6 | 0.5 | 651.9 |  |  |
|  | Calf raises | Both hands hold | normal stance | 12 | 30 | 1 | 30 | 60 | YES | 360 | 6 | 1.81 | 17.8 | 12.6 | 0.5 | 651.9 |  |  |
| **Total week 6** |  |  |  | **24** |  |  |  |  |  | **720** | **12** |  |  |  |  | **1303.9** | **3911.6** | **15755.2** |
| Week 7 | 90º Degree Squat | Crossed arms (Without hold) | wide stance | 9 | 30 | 1 | 30 | 30 | NO | 270 | 4.5 | 1.81 | 17.8 | 12.6 | 1 | 489.0 |  |  |
|  | Triceps dips | Hands on platform | - | 4 | 30 | 1 | 15 | 45 | NO | 60 | 1 | 1.81 | 17.8 | 12.6 | 0.33 | 108.7 |  |  |
|  | Calf raises | Both hands hold | normal stance | 9 | 30 | 1 | 30 | 30 | NO | 270 | 4.5 | 1.81 | 17.8 | 12.6 | 1 | 489.0 |  |  |
|  | Glute bridge | Crossed arms (Without hold) | on the platform | 5 | 30 | 1 | 15 | 45 | NO | 75 | 1.25 | 1.81 | 17.8 | 12.6 | 0.333 | 135.8 |  |  |
| **Total week 7** |  |  |  | **27** |  |  |  |  |  | **675** | **11.25** |  |  |  |  | **1222.4** | **3667.2** | **19422.4** |
| Week 8 | 90º Degree Squat | Crossed arms (Without hold) | wide stance | 10 | 30 | 1 | 30 | 30 | NO | 300 | 5 | 1.81 | 17.8 | 12.6 | 1 | 543.3 |  |  |
|  | Triceps dips | Hands on platform | - | 4 | 30 | 1 | 15 | 45 | NO | 60 | 1 | 1.81 | 17.8 | 12.6 | 0.33 | 108.7 |  |  |
|  | Calf raises | Both hands hold | normal stance | 10 | 30 | 1 | 30 | 30 | NO | 300 | 5 | 1.81 | 17.8 | 12.6 | 1 | 543.3 |  |  |
|  | Glute bridge | Crossed arms (Without hold) | on the platform | 5 | 30 | 1 | 15 | 45 | NO | 75 | 1.25 | 1.81 | 17.8 | 12.6 | 0.33 | 135.8 |  |  |
| **Total week 8** |  |  |  | **29** |  |  |  |  |  | **735** | **12.25** |  |  |  |  | **1331.0** | **3993.1** | **23415.5** |
| **Supplementary Table 4.** Month 2. Warm up of 10 minutes. BN: Bent knee; SWA: shoulder-width apart; Rep.: Repetitions; Freq: Frequency; Disp.: Displacement; Dur.: Duration; Vib.: Vibration; p: Peak; accel: acceleration, RMS: Root mean square. | | | | | | | | | | | | | | | | | | |

| **Week** | **Position** | **Hands and arms** | **Feet** | **Rep.** | **Freq. (Hz)** | **Displ. (mm)** | **Dur. (s)** | **Rest (s)** | **Active rest** | **Total vib. (s)** | **Total vib. (min)** | **P. accel (apeak) g** | **P. accel (apeak) m/s^2^** | **RMS Accel. (aRMS) m/s^2^** | **Density (Work/rest)** | **Dose (vibration (g)*time (s))** | **Total weekly dose** | **Acumulated dose** |
| --- | --- | --- | --- | --- | --- | --- | --- | --- | --- | --- | --- | --- | --- | --- | --- | --- | --- | --- |
| Week 9 | Split squat | Both hands hold | normal stance | 10 | 30 | 1 | 30 | 30 | NO | 300 | 5 | 1.81 | 17.8 | 12.6 | 1 | 543.3 |  |  |
|  | Bent knee Calf raises | Both hands hold | normal stance | 10 | 30 | 1 | 30 | 30 | NO | 300 | 5 | 1.81 | 17.8 | 12.6 | 1 | 543.3 |  |  |
|  | Kneeling push-up | Hands on platform SWA | out of the platform | 4 | 30 | 1 | 15 | 45 | NO | 60 | 1 | 1.81 | 17.8 | 12.6 | 0.33 | 108.7 |  |  |
|  | Glute bridge | Crossed arms (Without hold) | on the platform | 6 | 30 | 1 | 30 | 30 | NO | 180 | 3 | 1.81 | 17.8 | 12.6 | 1 | 326.0 |  |  |
| **Total week 9** |  |  |  | **30** |  |  |  |  |  | **840** | **14** |  |  |  |  | **1195.2** | **3585.7** | **27327.2** |
| Week 10 | Split squat | One hand hold | normal stance | 11 | 35 | 1 | 30 | 30 | NO | 330 | 5.5 | 2.46 | 24.2 | 17.1 | 1 | 813.4 |  |  |
|  | BN Calf raises | Both hands hold | normal stance | 11 | 35 | 1 | 30 | 30 | NO | 330 | 5.5 | 2.46 | 24.2 | 17.1 | 1 | 813.4 |  |  |
|  | Kneeling push-up | Hands on platform SWA | out of the platform | 4 | 30 | 1 | 30 | 30 | NO | 120 | 2 | 1.81 | 17.8 | 12.6 | 1 | 217.3 |  |  |
|  | Glute bridge | Crossed arms (Without hold) | on the platform | 6 | 30 | 1 | 30 | 30 | NO | 180 | 3 | 1.81 | 17.8 | 12.6 | 1 | 326.0 |  |  |
| **Total week 10** |  |  |  | **32** |  |  |  |  |  | **960** | **16** |  |  |  |  | **1844.1** | **5532.4** | **32859.6** |
| Week 11 | Split squat | Crossed arms (Without hold) | normal stance | 10 | 35 | 1 | 30 | 30 | NO | 300 | 5 | 2.46 | 24.2 | 17.1 | 1 | 739.5 |  |  |
|  | Plank | Forearms on the platform | out of the platform | 2 | 30 | 1 | 15 | 45 | NO | 30 | 0.5 | 1.81 | 17.8 | 12.6 | 0.33 | 54.3 |  |  |
|  | BN Calf raises | Both hands hold | normal stance | 10 | 35 | 1 | 30 | 30 | NO | 300 | 5 | 2.46 | 24.2 | 17.1 | 1 | 739.5 |  |  |
|  | Kneeling push ups | Hands on platform SWA | out of the platform | 3 | 30 | 1 | 30 | 30 | NO | 90 | 1.5 | 1.81 | 17.8 | 12.6 | 1 | 163.0 |  |  |
|  | Glute bridge | Crossed arms (Without hold) | on the platform | 5 | 30 | 1 | 30 | 30 | NO | 150 | 2.5 | 1.81 | 17.8 | 12.6 | 1 | 271.6 |  |  |
| **Total week 11** |  |  |  | **30** |  |  |  |  |  | **870** | **14.5** |  |  |  |  | **1967.9** | **5903.7** | **38763.3** |
| Week 12 | Split squat | Crossed arms (Without hold) | normal stance | 7 | 35 | 1 | 45 | 15 | NO | 315 | 5.25 | 2.46 | 24.2 | 17.1 | 3 | 776.4 |  |  |
|  | Plank | Forearms on the platform | out of the platform | 3 | 30 | 1 | 15 | 45 | NO | 45 | 0.75 | 1.81 | 17.8 | 12.6 | 0.33 | 81.5 |  |  |
|  | BN Calf raises | Both hands hold | normal stance | 7 | 35 | 1 | 45 | 15 | NO | 315 | 5.25 | 2.46 | 24.2 | 17.1 | 3 | 776.4 |  |  |
|  | Kneeling push ups | Hands on platform SWA | out of the platform | 4 | 30 | 1 | 30 | 30 | NO | 120 | 2 | 1.81 | 17.8 | 12.6 | 1 | 217.3 |  |  |
|  | Glute bridge | Crossed arms (Without hold) | on the platform | 5 | 30 | 1 | 30 | 30 | NO | 150 | 2.5 | 1.81 | 17.8 | 12.6 | 1 | 271.6 |  |  |
| **Total week 12** |  |  |  | **26** |  |  |  |  |  | **945** | **15.75** |  |  |  |  | **2123.3** | **6370.0** | **45133.3** |
| **Supplementary Table 5.** Month 3. Warm up of 10 minutes. BN: Bent knee; SWA: shoulder-width apart; Rep.: Repetitions; Freq: Frequency; Disp.: Displacement; Dur.: Duration; Vib.: Vibration; p: Peak; accel: acceleration, RMS: Root mean square. | | | | | | | | | | | | | | | | | | |

| **Week** | **Position** | **Hands and arms** | **Feet** | **Rep.** | **Freq. (Hz)** | **Displ. (mm)** | **Dur. (s)** | **Rest (s)** | **Active rest** | **Total vib. (s)** | **Total vib. (min)** | **Peak accel (apeak) g** | **Peak accel (apeak) m/s^2^** | **RMS Acceleration (aRMS) m/s^2^** | **Density (Work/rest)** | **Dose (vibration (g)*time (s))** | **Total weekly dose** | **Acumulated dose** |
| --- | --- | --- | --- | --- | --- | --- | --- | --- | --- | --- | --- | --- | --- | --- | --- | --- | --- | --- |
| Week 13 | Dynamic squat (90-120) | Both hands hold | normal stance | 9 | 30 | 2 | 45 | 15 | NO | 405 | 6.75 | 9 | 30 | 2 | 45 | 1466.9 |  |  |
|  | Plank | Forearms on the platform | out of the platform | 4 | 30 | 1 | 15 | 45 | NO | 60 | 1 | 4 | 30 | 1 | 15 | 108.7 |  |  |
|  | Step Lunge | Arms free | one on step and the other on platform | 5 | 30 | 1 | 30 | 30 | NO | 150 | 2.5 | 5 | 30 | 1 | 30 | 271.6 |  |  |
|  | Kneeling push ups^+^ | Hands on platform SWA | on a step | 4 | 30 | 1 | 30 | 30 | NO | 120 | 2 | 4 | 30 | 1 | 30 | 217.3 |  |  |
|  | Glute bridge | Crossed arms (Without hold) | on the platform | 5 | 30 | 1 | 30 | 30 | NO | 150 | 2.5 | 5 | 30 | 1 | 30 | 271.6 |  |  |
| **Total week 13** |  |  |  | **27** |  |  |  |  |  | **885** | **14.75** | **27** |  |  |  | **2336.1** | **7008.4** | **52141.6** |
| Week 14 | Dynamic squat (90-120) | Both hands hold | normal stance | 10 | 30 | 2 | 45 | 15 | NO | 450 | 7.5 | 10 | 30 | 2 | 45 | 1629.9 |  |  |
|  | Plank | Forearms on the platform | out of the platform | 4 | 30 | 1 | 20 | 40 | NO | 80 | 1.33 | 4 | 30 | 1 | 20 | 144.9 |  |  |
|  | Step Lunge | Arms free | one on step and the other on platform | 6 | 30 | 1 | 45 | 15 | NO | 270 | 4.5 | 6 | 30 | 1 | 45 | 489.0 |  |  |
|  | Kneeling push ups^+^ | Hands on platform SWA | on a step | 4 | 30 | 1 | 30 | 30 | NO | 120 | 2 | 4 | 30 | 1 | 30 | 217.3 |  |  |
|  | Glute bridge | Crossed arms (Without hold) | on the platform | 6 | 30 | 1 | 30 | 30 | NO | 180 | 3 | 6 | 30 | 1 | 30 | 326.0 |  |  |
| **Total week 14** |  |  |  | **30** |  |  |  |  |  | **1100** | **18.33** | **30** |  |  |  | **2807.0** | **8420.9** | **60562.5** |
| Week 15 | Dynamic squat (90-120) | Crossed arms (Without hold) | wide stance | 11 | 30 | 2 | 45 | 15 | NO | 495 | 8.25 | 11 | 30 | 2 | 45 | 1792.8 |  |  |
|  | Plank | Forearms on the platform | out of the platform | 4 | 30 | 1 | 20 | 40 | NO | 80 | 1.33 | 4 | 30 | 1 | 20 | 144.9 |  |  |
|  | Step Lunge | Arms free | one on step and the other on platform | 7 | 30 | 1 | 45 | 15 | NO | 315 | 5.25 | 7 | 30 | 1 | 45 | 570.4 |  |  |
|  | Push ups | Hands on platform SWA | out of the platform | 5 | 30 | 1 | 20 | 40 | NO | 100 | 1.67 | 5 | 30 | 1 | 20 | 181.1 |  |  |
|  | Glute bridge | Crossed arms (Without hold) | on the platform | 6 | 30 | 1 | 30 | 30 | NO | 180 | 3 | 6 | 30 | 1 | 30 | 326.0 |  |  |
| **Total week 15** |  |  |  | **33** |  |  |  |  |  | **1170** | **19.5** |  |  |  |  | **3015.2** | **9045.7** | **69608.2** |
| Week 16 | Dynamic squat (90-120) | Crossed arms (Without hold) | wide stance | 11 | 30 | 2 | 45 | 15 | NO | 495 | 8.25 | 11 | 30 | 2 | 45 | 1792.8 |  |  |
|  | Plank | Forearms on the platform | out of the platform | 4 | 30 | 1 | 20 | 40 | NO | 80 | 1.33 | 4 | 30 | 1 | 20 | 144.9 |  |  |
|  | Step Lunge | Arms free | one on step and the other on platform | 7 | 30 | 1 | 45 | 15 | NO | 315 | 5.25 | 7 | 30 | 1 | 45 | 570.4 |  |  |
|  | Push ups | Hands on platform SWA | out of the platform | 5 | 30 | 1 | 20 | 40 | NO | 100 | 1.67 | 5 | 30 | 1 | 20 | 181.1 |  |  |
|  | Glute bridge | Crossed arms (Without hold) | on the platform | 6 | 30 | 1 | 45 | 15 | NO | 270 | 4.5 | 6 | 30 | 1 | 45 | 489.0 |  |  |
| **Total week 16** |  |  |  | **33** |  |  |  |  |  | **1260** | **21** | **33** |  |  |  | **3178.2** | **9534.6** | **79142.8** |
| **Supplementary Table 6.** Month 4. Warm up of 10 minutes. BN: Bent knee; SWA: shoulder-width apart; Rep.: Repetitions; Freq: Frequency; Disp.: Displacement; Dur.: Duration; Vib.: Vibration; p: Peak; accel: acceleration, RMS: Root mean square.+ Feet on a step that is the same height as the WBV so that the push-ups are performed totally horizontal | | | | | | | | | | | | | | | | | | |

| **Supplementary table 7:** Checklist of information regarding the WBV | | |
| --- | --- | --- |
| *Item* | *Short description* |  |
| 1 | Device specifications | Power Plate® Pro5 |
| 2 | If applicable: platform constructions (e.g., mounting a chair on it) | Not applicable |
| **Information about the vibration** | |  |
| *Item* | *Short description* |  |
| 3 | The type of vibration: spatial and temporal characteristics (e.g., vertical or side-alternating) | Vertical |
| 4 | The vibration parameters: definitions and parameter settings used | Specified in Table 1, and Supplementary tables 2, 3, 4 and 5 |
| 5 | Whether and how the vibration parameters were verified | **3.4.3.4. Whole body vibration device**  *“The specific device, presents a constant frequency and magnitude and the vibration parameters have been previously tested in our laboratory”* |
| 6 | For side-alternating vibrations: the location on the vibration platform where the accelerometer was placed to measure magnitude | Not applicable |
| 7 | Whether frequency and magnitude were constant or modulated | **3.4.3.4. Whole body vibration device**  *“The specific device, presents a constant frequency and magnitude and the vibration parameters have been previously tested in our laboratory”* |
| **Information about the administration** | |  |
| *Item* | *Short description* |  |
| 8 | The posture or body position of the subject and whether it was changed during the intervention (static versus dynamic exercise) | Figure 2, and supplementary tables 2, 3, 4 and 5 |
| 9 | The position of the feet of the subjects on the platform during the vibration and how the feet were loaded (e.g., on midfoot or on forefoot with heel lifted off) | Supplementary tables 2, 3, 4 and 5. 3.4.3.2. Session structure “*All exercises will be performed with the whole foot sole (both forefoot and heels) touching the platform except for the calf raises and bent knee calf raises that were performed without contact of the heel with the platform*” |
| 10 | If and how skidding of feet was prevented | **3.4.3.2. Session structure**  “*All participants will wear socks while performing the training which in contact with the mat provided by power-plate avoids skidding of participants*”. |
| 11 | If and how vibration transmission to the head was prevented | **3.4.3.2. Session structure**  “*As specified in* ***Figure 2,*** *all exercise will be performed with flexion of at least one lower limb joint to avoid vibration transmission to the head.”* |
| 12 | If a handrail was available/used | **3.4.3.2. Session structure**  *“During the first week of each month new exercises will be implemented and participants will be allowed to hold on to the WBV platform handrail with both hands to perform the exercises. The next week only one hand will be used and participants will progress to perform the exercise without holding to the handrail in the last weeks of each month as explained in* ***supplementary tables 2, 3, 4 and 5.”*** |
| 13 | The position of the hands during the WBV and if the hands were directly subjected to the vibration | Supplementary tables 2, 3, 4 and 5. |
| 14 | If applicable, the parts of the subjects’ body which were most subjected to vibration (e.g., predominantly the feet) | Figure 2 and supplementary tables 2, 3, 4 and 5. |
| 15 | Whether and, if applicable, what tools/aids were used during the vibration (e.g., type and size of dumbbells or resistance bands) | **3.4.3.2. Session structure**  *“No dumbbells or resistance bands will be used while performing the exercises on the WBV device.”* |
| 16 | General exercise parameters (e.g., duration, number of bouts, rest intervals) | Table 1 and Supplementary tables 2, 3, 4 and 5. |
| **Information in general protocol** | |  |
| *Item* | *Short description* |  |
| 17 | The setting of the WBV sessions/intervention (e.g., hospital, gym, or at home) and the time of day the sessions took place | **3.4.3.1. Supervision and adherence**  *“The training will be free for all participants and will be performed at our laboratory (GENUD-Lab University of Zaragoza, Spain) without a home-based component”*  **3.4.3.3. Training frequency, intensity and duration**  *“Participants will be able to choose the time of day and day of the week to perform them with the only condition that at least 24 hours have to pass between sessions and the training cannot be performed in three consecutive days.”* |
| 18 | Presence of a trainer (face to face/online) during the WBV sessions | **3.4.3.1. Supervision and adherence**  *“All the training sessions will be supervised and conducted with one-to-one supervision by a sport scientist who has previous experience working with WBV platforms and will guide the participant through the training session”.* |
| 19 | The instructions given to the subject before the WBV session | **3.4.3.2. Session structure**  *“At the beginning of the WBV session, the supervisor will explain the posture/exercises to be executed during the WBV session.”* |
| 20 | Preparatory exercises or warm-up prior to the vibration (type and duration of exercises) | **3.4.3.2. Session structure**  *“The warm-up will consist of cycling at a frequency between 60 to 70 revolutions per minute performed on an electromagnetic cycle ergometer (Ergoline Viasprint150p).*” |
| 21 | The subjects’ footwear (shoes, socks, barefoot) during the vibration, with a detailed description | **3.4.3.2. Session structure**  *“All participants will wear socks while performing the training which in contact with the mat provided by power-plate avoids skidding of participants.”* |
| 22 | If applicable: characteristics control/sham condition or intervention | NOT APPLICABLE |
| 23 | The moment at which the outcome measures were assessed: during, before, and/or after the vibration; the time between begin/end of exposure/exercise/session and assessment(s) | **3.1. Study Design, Protocol Registration and Reporting**  Figure 1 |
| **Information about the subjects** | |  |
| *Item* | *Short description* |  |
| 24 | General characteristics of the subjects | - - - 1. ***Inclusion criteria***   “*People that are going to undergo a sleeve gastrectomy surgery with a body mass index above 40 kg/m^2^ and less than 180 kg in weight, 2) With an age between 18 and 50 y*” |
| 25 | The subjects’ previous experience with WBV | NOT APPLICABLE |
| 26 | Acute, short term, or long-term side effects of the vibration exercise | **3.4.3.1. Supervision and adherence**  “*Pain and adverse effects during the session.“* |
